# Efficacy and Safety of Oral Prednisolone and Budesonide MMX for Outpatient Induction Therapy in Active Ulcerative Colitis: A Multicenter Retrospective Cohort Study

**DOI:** 10.3390/jcm15135115

**Published:** 2026-07-01

**Authors:** Kentaro Kojima, Jun Takada, Keisuke Iwata, Kiichi Otani, Naoya Masuda, Hiroki Taniguchi, Koji Yamashita, Noritaka Ozawa, Sachiyo Onishi, Masaya Kubota, Takashi Ibuka, Kenji Yamazaki, Masahito Shimizu

**Affiliations:** 1 Department of Gastroenterology and Internal Medicine, Gifu University Graduate School of Medicine, 1-1 Yanagido, Gifu 501-1194, Japangogo_nishi0924@yahoo.co.jp (S.O.);; 2 Department of Gastroenterology, Gifu Prefectural General Medical Center, 4-6-1 Noishiki, Gifu 500-8717, Japan

**Keywords:** ulcerative colitis, budesonide, prednisolone, remission induction, adverse effects

## Abstract

**Background/Objectives:** Oral prednisolone (PSL) and budesonide multi-matrix (BUD-MMX) are used to induce remission in ulcerative colitis (UC); however, their therapeutic positioning remains unclear. Comparative data from real-world practices are limited. This study assessed the efficacy and safety of PSL and BUD-MMX in patients with active UC. **Methods:** Consecutive outpatients with UC initiated on oral PSL or BUD-MMX at two tertiary referral centers were included. The primary outcome was clinical remission at week 8, defined as a partial Mayo score (PMS) ≤ 1 with a rectal bleeding subscore of 0. Secondary outcomes included clinical response and safety. Pre-specified subgroup analyses were performed according to baseline disease activity. **Results:** Sixty PSL-treated and 40 BUD-MMX-treated patients were evaluated at week 8. Clinical remission was achieved in 65.0% and 55.0% of the PSL and BUD-MMX groups, respectively, whereas a clinical response was observed in 75.0% and 62.5%, respectively. No patient with a baseline PMS of 2–3 was initiated on PSL. Among patients with a baseline PMS of 4–5, the remission and response rates at week 8 were similar between the groups. In contrast, remission in patients with a baseline PMS ≥ 6 was numerically lower in the BUD-MMX group. Treatment escalation rates were comparable in the overall cohort, whereas adverse events were more frequent in the PSL group (23.3% vs. 2.4%). **Conclusions:** Treatment selection between PSL and BUD-MMX appeared to reflect baseline disease activity, with a partial overlap in the moderate clinical range. BUD-MMX may be a reasonable initial option for selected patients.

## 1. Introduction

Ulcerative colitis (UC) is a chronic relapsing inflammatory disorder of the colon characterized by periods of active inflammation and remission [1]. Achieving timely induction of remission is crucial not only for symptom control but also for preventing disease progression, reducing hospitalization, and improving long-term outcomes. Current therapeutic strategies increasingly emphasize treat-to-target approaches aimed at early clinical remission and mucosal healing [2].

In patients with mild-to-moderate disease activity, 5-aminosalicylates (5-ASA) are recommended as the first-line therapy [3,4]. In addition to 5-ASA, several approved therapeutic options are available for UC, including corticosteroids, immunomodulators, biologic agents, and small-molecule agents. These therapies have improved disease control, but treatment selection may be affected by drug-specific factors, including delayed onset of action, systemic adverse effects, infection risk, and treatment burden. Among patients with mild-to-moderate disease activity, some do not achieve adequate disease control with 5-ASA alone and require treatment escalation. In this setting, oral corticosteroids remain an important next-step option. Prednisolone (PSL), a systemic corticosteroid, is effective for inducing remission in active UC; however, its use is associated with well-recognized adverse effects, including infections, metabolic complications, and psychiatric symptoms [3,4,5]. Consequently, current treatment strategies aim to minimize systemic corticosteroid exposure and avoid repeated steroid reintroduction whenever feasible [6].

As an alternative to systemic corticosteroids, budesonide multi-matrix (BUD-MMX) was developed as a locally acting oral corticosteroid designed to deliver the drug throughout the colon while reducing systemic bioavailability [7,8]. BUD-MMX has been approved for the treatment of mild-to-moderate UC based on randomized controlled trials and is now used in routine practice [9,10]. Owing to its high first-pass hepatic metabolism and low systemic exposure, BUD-MMX is expected to provide a more favorable safety profile compared with systemic corticosteroids [11]. Current guidelines generally position BUD-MMX for mild-to-moderate disease activity, whereas systemic corticosteroids are more commonly used for patients with moderate to severe disease activity [3,4]. However, this therapeutic distinction is supported mainly by placebo-controlled trials and indirect evidence rather than direct comparative studies. Moreover, earlier comparative data between PSL and budesonide did not include a colonic-release budesonide formulation [4]. In routine outpatient practice, the therapeutic positioning of PSL and BUD-MMX may therefore partly overlap, particularly in patients with moderate disease activity. In such cases, treatment selection is influenced by multiple factors including disease extent, prior treatment history, and safety considerations.

The clinical positioning of PSL and BUD-MMX in the management of active UC has not been well characterized, and comparative real-world data on short-term clinical outcomes between these agents remain limited [12,13]. Therefore, we conducted a multicenter retrospective cohort study of outpatients with active UC who received oral PSL or BUD-MMX to evaluate real-world treatment patterns as well as short-term effectiveness and safety.

## 2. Materials and Methods

### 2.1. Study Design and Setting

This retrospective observational study was conducted at two tertiary referral centers in Japan. Eligible patients were identified from the outpatient records during the study period. Patients who started PSL between January 2015 and December 2025, and those who started BUD-MMX between September 2023 and December 2025 were included. The different enrollment periods reflected the timing of BUD-MMX availability in Japan, where it became clinically available in September 2023, following regulatory approval in June 2023. The index date was defined as the date of the first PSL or BUD-MMX prescription. Only the first induction episode for each treatment was included in the analysis for each patient.

### 2.2. Patient Selection

Consecutive outpatients with UC initiated on oral PSL or BUD-MMX as induction therapy during the study period were eligible for inclusion. The diagnosis of UC was established based on standard clinical, endoscopic, and histological criteria [14]. Active disease at baseline was defined as a partial Mayo score (PMS) ≥ 2 [15]. In the PSL group, patients with no prior exposure to PSL were included. Patients with no prior exposure to BUD-MMX were included in the BUD-MMX group; prior PSL use was permitted in this group. Advanced therapies (AT) were defined as biological agents and Janus kinase (JAK) inhibitors. Patients were excluded if baseline PMS was ≤1, if AT was started on the same day as the study drug, or if the study drug was prescribed for on-demand or intermittent use rather than as a planned induction course.

### 2.3. Data Collection

Clinical, endoscopic, and laboratory data were retrospectively collected from the electronic medical records of the participating institutions. Baseline variables included patient demographics (age, sex, and body mass index), disease duration, disease extent, baseline PMS, Mayo endoscopic subscore when available, and laboratory parameters, including C-reactive protein (CRP), albumin, white blood cell count, lymphocyte count, hemoglobin, and platelet count. Data on previous treatment history, including systemic corticosteroids, immunomodulators, and AT, were also collected, along with concomitant medications at treatment initiation. For PSL-treated patients, the dose-reduction schedule was not protocolized and was determined by the treating physician according to clinical response and safety considerations. The duration of study drug treatment was calculated from treatment initiation to treatment discontinuation based on prescription records. Follow-up assessments were performed at approximately 4 and 8 weeks after treatment initiation based on outpatient visit records. Adverse events during the 8-week observation period were also recorded.

### 2.4. Outcomes and Definitions

The primary outcome was clinical remission at week 8, defined as a PMS ≤ 1 with a rectal bleeding subscore of 0 [16]. Secondary outcomes included clinical remission at week 4, clinical response at weeks 4 and 8, and treatment escalation and adverse events within 8 weeks. Clinical response was defined as a decrease of ≥2 points in PMS from baseline. Patients who required treatment escalation were classified as nonresponders at the respective time points. Treatment escalation was defined as the intensification of systemic corticosteroid therapy (initiation of oral or intravenous corticosteroids or dose escalation of oral corticosteroids) or initiation of AT. Subgroup analyses were performed according to baseline PMS strata (PMS 2–3, PMS 4–5, and PMS ≥ 6). In patients with baseline PMS 4–5, exploratory logistic regression analyses were performed to evaluate the association between treatment and week 8 clinical remission. Longitudinal changes in disease activity and laboratory biomarker levels were also assessed in all patients who achieved week 8 clinical remission.

### 2.5. Statistical Analysis

Continuous variables are expressed as medians with interquartile ranges (IQRs) and categorical variables as counts and percentages. Group comparisons were performed using the Wilcoxon rank-sum test for continuous variables and Fisher’s exact test for categorical variables. Clinical outcomes were compared between the PSL and BUD-MMX groups in the overall cohort and in a subgroup of patients with baseline PMS 4–5. To assess the potential influence of temporal bias related to the different enrollment periods, we performed an exploratory sensitivity analysis by restricting the PSL group to patients who initiated PSL from September 2023. Baseline characteristics, clinical outcomes, treatment escalation, and adverse events were compared between this contemporaneous PSL subgroup and the BUD-MMX group using the same statistical methods as in the main analysis. Week 8 clinical remission rates were also summarized according to baseline PMS strata (PMS 2–3, PMS 4–5, and PMS ≥ 6). In the subgroup with a baseline PMS of 4–5, unadjusted and CRP-adjusted logistic regression models were used to evaluate the association between treatment and week 8 clinical remission, and odds ratios (ORs) with 95% confidence intervals (CIs) were calculated. Among the patients who achieved week 8 clinical remission, within-group changes in clinical and laboratory parameters over time were assessed using the Wilcoxon signed-rank test. All statistical tests were two-sided, and a *p* value < 0.05 was considered statistically significant. All analyses were performed using the R software (version 4.5.1; R Foundation for Statistical Computing, Vienna, Austria).

## 3. Results

### 3.1. Patients and Baseline Characteristics

**Figure 1:**
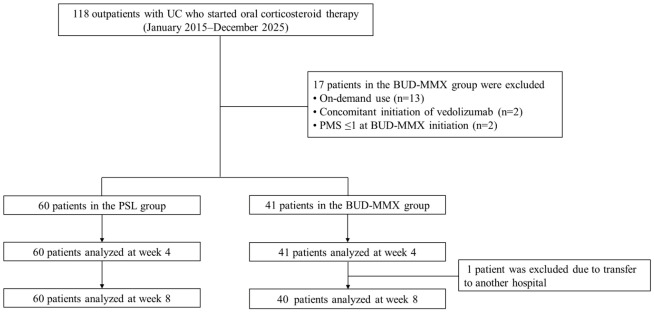
Study flow diagram. Flow diagram of patient selection and the numbers of patients included in the baseline, week 4, and week 8 analyses in the PSL and BUD-MMX groups. UC, ulcerative colitis; PSL, prednisolone; BUD-MMX, budesonide MMX; PMS, partial Mayo score.

A total of 118 outpatients with UC initiated treatment during the study period, including 60 who initiated PSL and 58 who initiated BUD-MMX (Figure 1). Sixty patients in the PSL group were included in the analysis. In the BUD-MMX group, 17 patients were excluded because of on-demand or intermittent use (n = 13), concomitant initiation of vedolizumab (n = 2), or a baseline PMS ≤ 1 (n = 2), leaving 41 patients for the baseline and week 4 analyses. At week 8, one patient in the BUD-MMX group was not evaluable because of transfer to another hospital, resulting in 60 evaluable patients in the PSL group and 40 in the BUD-MMX group.

The baseline characteristics are summarized in Table 1. Patients in the BUD-MMX group had a significantly longer disease duration than those in the PSL group (*p* < 0.001). The inflammatory marker levels and disease activity were higher in the PSL group, with significantly higher white blood cell counts (*p* = 0.009), CRP levels (*p* < 0.001), and PMS (*p* < 0.001). Previous exposure to immunomodulators or AT was more frequent in the BUD-MMX group (both *p* < 0.001). The concomitant use of immunomodulators (*p* = 0.017) and AT (*p* < 0.001) was also more common in the BUD-MMX group. No other baseline characteristics differed significantly between the groups. The median initial PSL dose was 30 mg/day (IQR, 30–40 mg/day), whereas all patients in the BUD-MMX group received 9 mg/day. The median treatment duration was 80.5 days (IQR, 68–106 days) in the PSL group and 56 days (IQR, 48–66 days) in the BUD-MMX group.

### 3.2. Overall Clinical Remission and Response Rates

**Figure 2:**
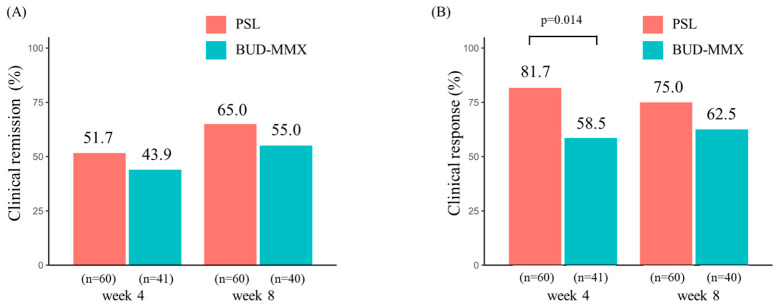
Overall clinical outcomes at weeks 4 and 8. (**A**) Clinical remission rates and (**B**) clinical response rates at weeks 4 and 8 in the PSL and BUD-MMX groups. Numbers below the bars indicate the number of evaluable patients at each time point. PSL, prednisolone; BUD-MMX, budesonide MMX.

The clinical remission and response rates at weeks 4 and 8 are shown in Figure 2. At week 4, clinical remission was achieved in 51.7% (31/60) of the patients in the PSL group and 43.9% (18/41) of those in the BUD-MMX group (*p* = 0.544), whereas a clinical response was observed in 81.7% (49/60) and 58.5% (24/41), respectively (*p* = 0.014). At week 8, clinical remission was achieved in 65.0% (39/60) of the patients in the PSL group and 55.0% (22/40) of those in the BUD-MMX group (*p* = 0.403), whereas clinical response was observed in 75.0% (45/60) and 62.5% (25/40), respectively (*p* = 0.191).

### 3.3. Treatment Escalation and Adverse Events in the Overall Cohort

Treatment escalation and adverse events within 8 weeks in the overall cohort are shown in Table 2. Treatment escalation occurred in 26.7% (16/60) of the PSL group and 26.8% (11/41) of the BUD-MMX group (*p* = 1.000). Systemic corticosteroid intensification occurred in 15.0% (9/60) of the PSL group and 7.3% (3/41) of the BUD-MMX group, and AT initiation in 11.7% (7/60) and 19.5% (8/41), respectively; neither difference was statistically significant. Adverse events were significantly more frequent in the PSL group than in the BUD-MMX group (23.3% vs. 2.4%; *p* = 0.004). The adverse events in the PSL group included neurological, psychiatric, dermatological, and infectious events, whereas only one infectious event was observed in the BUD-MMX group. Details of adverse events are shown in Supplementary Table S1. One serious adverse event (spinal cord infarction) occurred in the PSL group, whereas no serious adverse events were observed in the BUD-MMX group.

### 3.4. Exploratory Sensitivity Analysis Restricted to the PSL Subgroup from September 2023

In this exploratory sensitivity analysis, 31 patients in the contemporaneous PSL subgroup and 41 patients in the BUD-MMX group were included. Although some baseline differences remained between the groups, clinical outcomes were generally consistent with those of the main analysis. Week 8 clinical remission was achieved in 20 of 31 patients (64.5%) in the PSL subgroup and 22 of 40 patients (55.0%) in the BUD-MMX group (*p* = 0.472), and clinical response was achieved in 23 of 31 patients (74.2%) and 25 of 40 patients (62.5%), respectively (*p* = 0.320). Treatment escalation rates within 8 weeks were also similar between the groups (25.8% vs. 26.8%, *p* = 1.000), as were AT initiation rates (19.4% vs. 19.5%, *p* = 1.000). The results of this sensitivity analysis are shown in Supplementary Table S2.

### 3.5. Week 8 Clinical Remission According to Baseline Disease Activity

**Figure 3:**
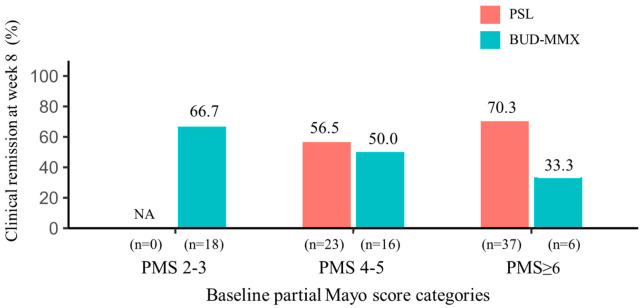
Clinical remission rates at week 8 according to baseline partial Mayo score categories. Bar graph showing week 8 clinical remission rates in the PSL and BUD-MMX groups stratified by baseline partial Mayo score categories. Numbers below the bars indicate the number of evaluable patients in each subgroup. PSL, prednisolone; BUD-MMX, budesonide MMX; PMS, partial Mayo score; NA, not applicable.

The clinical remission rates at week 8 according to the baseline PMS categories are shown in Figure 3. In the PMS 2–3 subgroup, no patient with baseline PMS 2–3 initiated PSL, whereas remission was achieved in 66.7% (12/18) of those who initiated BUD-MMX. Among patients with PMSs 4–5, the remission rates were 56.5% (13/23) in the PSL group and 50.0% (8/16) in the BUD-MMX group (*p* = 0.752). In patients with PMS ≥ 6, remission was observed in 70.3% (26/37) of the PSL group and 33.3% (2/6) of the BUD-MMX group.

### 3.6. Baseline Characteristics, Clinical Outcomes, and Logistic Regression Analysis in Patients with Baseline PMS 4–5

The baseline characteristics of patients with baseline PMS 4–5 are summarized in Table 3. Similar to the overall cohort, CRP levels were significantly higher in the PSL group than in the BUD-MMX group (*p* = 0.013). Previous immunomodulator and concomitant AT use were more frequent in the BUD-MMX group, whereas other baseline characteristics were broadly comparable between the groups. The clinical outcomes, treatment escalation, and adverse events in this subgroup are shown in Table 4. At week 4, clinical remission was achieved in 47.8% (11/23) of the PSL group and 43.8% (7/16) of the BUD-MMX group (*p* = 1.000), while clinical response was observed in 69.6% (16/23) and 68.8% (11/16), respectively (*p* = 1.000). At week 8, a clinical response was observed in 60.9% (14/23) of the PSL group and 56.2% (9/16) of the BUD-MMX group (*p* = 1.000). Treatment escalation occurred in 39.1% (9/23) of the PSL group and 37.5% (6/16) of the BUD-MMX group (*p* = 1.000), and adverse events were observed in 21.7% (5/23) and 0% (0/16), respectively (*p* = 0.066). Exploratory logistic regression analyses of patients with baseline PMS 4–5 showed no significant association between treatment and week 8 clinical remission (Table 5). In the unadjusted model, BUD-MMX was not significantly associated with week 8 clinical remission compared with PSL (OR 0.77, 95% CI 0.21–2.77, *p* = 0.688). This association remained non-significant in the CRP-adjusted model (OR 0.55, 95% CI 0.14–2.14, *p* = 0.385).

### 3.7. Longitudinal Changes Among Patients Who Achieved Week 8 Clinical Remission

**Figure 4:**
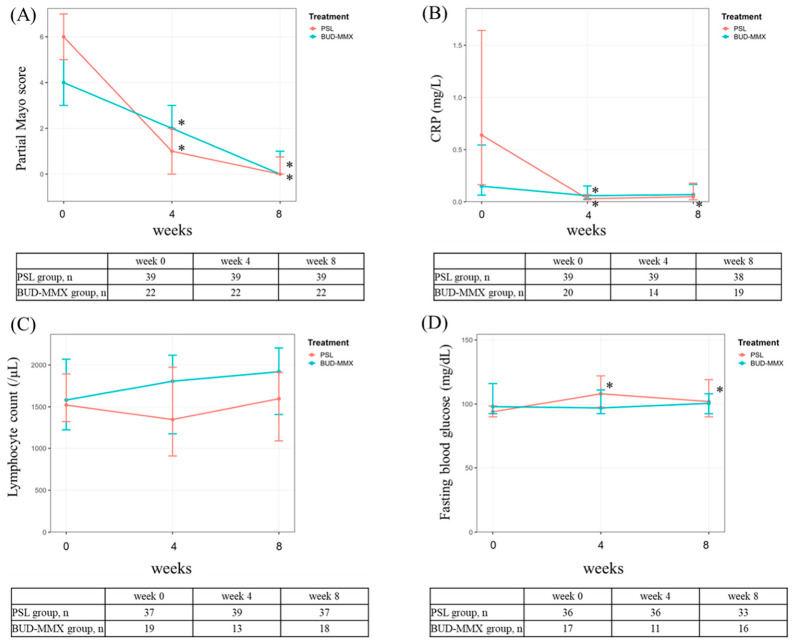
Longitudinal changes in clinical activity and selected laboratory markers among patients who achieved clinical remission at week 8. (**A**) Partial Mayo score, (**B**) C-reactive protein, (**C**) lymphocyte count, and (**D**) fasting blood glucose in the PSL and BUD-MMX groups at baseline and weeks 4 and 8. Asterisks indicate *p* < 0.05 versus baseline within the same treatment group. PSL, prednisolone; BUD-MMX, budesonide MMX; CRP, C-reactive protein.

In both groups, PMS was significantly lower at weeks 4 and 8 than at baseline (all *p* < 0.001) (Figure 4A). CRP levels decreased significantly at weeks 4 and 8 in the PSL group (both *p* < 0.001). In the BUD-MMX group, CRP levels were already low at baseline and remained low at weeks 4 and 8 [0.23 mg/dL (IQR, 0.08–0.55), 0.05 mg/dL (IQR, 0.03–0.10), and 0.06 mg/dL (IQR, 0.02–0.18), respectively]. The reduction from baseline was statistically significant at week 4 (*p* = 0.011), but did not reach statistical significance at week 8 (*p* = 0.074) (Figure 4B). The lymphocyte counts showed no significant longitudinal changes in either group (Figure 4C). Fasting blood glucose levels increased significantly at weeks 4 and 8 in the PSL group (*p* = 0.012 and *p* = 0.018, respectively), with no significant changes in the BUD-MMX group (Figure 4D).

## 4. Discussion

This two-center retrospective study evaluated the effectiveness and safety of oral PSL and BUD-MMX as initial induction therapies for outpatients with active UC. In this real-world cohort, the two treatments were selected in different clinical contexts according to the baseline disease activity. Among patients with a baseline PMS of 4–5, in whom the therapeutic positioning of the two agents may overlap, the short-term effectiveness was broadly similar between PSL and BUD-MMX. In terms of safety, adverse events were more frequent in the PSL group.

Current clinical guidelines recommend BUD-MMX as a nonsystemic corticosteroid option for mild-to-moderately active UC, whereas oral PSL is generally used in patients with moderate-to-severe disease activity [3,4,14]. In routine clinical practice, treatment selection is largely influenced by the overall clinical severity. Consistent with this, in the present study, BUD-MMX tended to be selected for patients with lower disease activity, whereas PSL was used more often for those with higher disease activity. Nevertheless, the therapeutic positions of these agents partially overlap in patients with moderate disease activity. In our study, patients with a baseline PMS of 4–5 were considered representative of this clinical range, and short-term outcomes were similar between the PSL and BUD-MMX groups within this subgroup.

The efficacy of BUD-MMX for the induction of remission in mild-to-moderate UC has been established in randomized controlled trials, including the CORE I and CORE II studies, as well as in subsequent real-world reports [9,10,17,18,19,20]. However, direct comparison of remission rates across studies requires caution because the endpoint definitions differ substantially. In pivotal trials, remission was defined using a stringent combination of clinical and endoscopic criteria, whereas many observational studies have used symptom-based clinical outcomes. In our cohort, the week 8 remission rate of 55.0% in the BUD-MMX group was consistent with previous real-world observations [18,19,21,22].

Oral PSL has long been used as conventional induction therapy for active UC because of its well-recognized efficacy in inducing remission. However, its use is limited by the burden of systemic adverse effects, and minimizing corticosteroid exposure remains an important therapeutic goal [23,24]. Direct comparative evidence between oral PSL and BUD-MMX is limited [12,13]. In our cohort, the two treatment groups were not fully comparable at baseline. The BUD-MMX group had lower baseline CRP levels and PMS but also had a longer disease duration and more frequent prior exposure to immunomodulators and AT. These findings suggest that the comparison between PSL and BUD-MMX should be interpreted in the context of patient selection, rather than as a direct comparison of drug superiority. Descriptively, PSL showed numerically higher clinical remission and response rates than BUD-MMX in the overall cohort, including a significantly higher clinical response rate at week 4, whereas treatment escalation rates within 8 weeks were similar between the two groups.

Stratification by baseline disease activity provides an additional clinical context because treatment selection in routine practice is closely linked to baseline severity. In patients with a baseline PMS of 4–5, the outcomes were similar between PSL and BUD-MMX, suggesting that BUD-MMX may be an induction option for selected outpatients within this moderate clinical range. None of the patients with PMS 2–3 received PSL, likely reflecting routine practice, in which systemic corticosteroids are generally not selected for patients with relatively mild baseline activity. In contrast, in the subgroup with a baseline PMS ≥ 6, the remission rate with BUD-MMX was numerically lower than that with PSL. Although this finding should be interpreted with caution due to the small number of BUD-MMX-treated patients in this subgroup, it suggests that PSL may remain an important induction option for patients with higher baseline disease activity. This observation is broadly consistent with findings from a British real-world comparative study in which baseline disease activity was relatively high, with a mean PMS of approximately 6, and less favorable early outcomes were observed with BUD-MMX than with PSL [12].

The longitudinal analysis of patients who achieved week 8 remission provided additional insight into the early treatment response. In both groups, the PMS and CRP levels improved significantly by week 4. These findings suggest that early improvement may be achieved with either treatment. In the BUD-MMX group, the absence of a statistically significant CRP reduction at week 8 does not necessarily indicate attenuation of treatment effect, because CRP levels were already low at baseline and remained low during follow-up, leaving only a limited absolute range for further reduction. Previous studies indicate that oral corticosteroids generally induce a clinical response within 1–4 weeks in UC [25]. While the STRIDE-II framework suggests that locally acting steroids may require somewhat longer than oral systemic corticosteroids to achieve clinical remission [2]. BUD-MMX was associated with early clinical improvement in our cohort, although the clinical response at week 4 was more frequent in the PSL group.

Safety considerations are also important when interpreting the clinical relevance of these findings. Although PSL remains an effective treatment option for active UC, systemic corticosteroids are associated with a broad range of adverse effects. In our cohort, adverse events occurred more frequently in the PSL group than in the BUD-MMX group, and those in the PSL group involved several categories, including neurologic, psychiatric, dermatologic, and infectious events, whereas only one infectious event was recorded in the BUD-MMX group. In addition, longitudinal changes in laboratory parameters did not suggest obvious systemic metabolic or hematological effects of BUD-MMX during follow-up. These observations are consistent with previous reports suggesting that second-generation corticosteroids are associated with fewer corticosteroid-related adverse events than conventional corticosteroids [26,27]. Collectively, these findings support the clinical value of BUD-MMX as a reasonable initial induction option for selected patients with moderate clinical activity, particularly in the clinical range in which short-term outcomes appear to be similar to those of PSL.

This study had several strengths and limitations. Its strengths include a real-world multicenter design and evaluation of the treatment patterns, effectiveness, and safety of PSL and BUD-MMX in outpatient induction therapy for active UC. However, this study has some limitations. First, due to the retrospective design, treatment selection was based on clinical judgment, and indication bias could not be excluded. Second, baseline differences between the groups may have resulted in residual confounding factors. In addition, the different enrollment periods of the PSL and BUD-MMX groups may have introduced a temporal bias related to changes in treatment practices over time. Although we performed an exploratory sensitivity analysis restricted to the PSL subgroup from September 2023, temporal bias could not be fully eliminated because baseline differences remained. Moreover, cumulative oral PSL exposure could not be accurately calculated because detailed dose-reduction schedules were not uniformly available. Third, the sample size was limited, especially for the subgroup analyses. Finally, although baseline endoscopic assessment was available in a subset of patients, follow-up endoscopic evaluation was not routinely performed. Therefore, PMS-defined clinical remission should be interpreted as short-term clinical effectiveness, but not as evidence of endoscopic remission or mucosal healing, because clinical symptoms may not fully reflect residual endoscopic inflammation. Prospective studies are required to confirm these findings and further clarify the clinical roles of PSL and BUD-MMX in active UC.

## 5. Conclusions

In conclusion, our multicenter real-world study provides insights into the clinical positioning of BUD-MMX and PSL in outpatient induction therapy for active UC. BUD-MMX may be a reasonable initial induction option for selected outpatients with moderate clinical activity, whereas PSL may still have an important role in the management of patients with higher disease activity.

## Figures and Tables

**Table 1:** Baseline characteristics of the overall cohort.

Characteristic	Overall (n = 101)	PSL (n = 60)	BUD-MMX (n = 41)	*p* Value
Male sex	54 (53.5)	30 (50.0)	24 (58.5)	0.424
Age (years)	50 (30, 64)	49 (30, 60)	54 (30, 66)	0.655
Duration of disease (months)	33 (7, 85)	18 (3, 55)	77 (27, 134)	<0.001
Disease extent				0.463
Proctitis	6 (5.9)	5 (8.3)	1 (2.4)	
Left-sided colitis	30 (29.7)	17 (28.3)	13 (31.7)	
Pancolitis	65 (64.4)	38 (63.3)	27 (65.9)	
Body mass index (kg/m^2^)	21.6 (19.5, 24.2)	21.3 (19.4, 24.9)	21.6 (19.6, 23.9)	0.768
White blood cell count (/μL)	6910 (5435, 8550)	7330 (6053, 9305)	6040 (5190, 7275)	0.009
Lymphocytes (/μL)	1540 (1266, 1917)	1522 (1324, 1894)	1582 (1225, 2070)	0.869
Hemoglobin (g/dL)	13.0 (11.5, 13.8)	13.0 (11.5, 13.8)	13.0 (11.3, 13.9)	0.641
Platelet count (×10^3^/μL)	287 (241, 346)	302 (243, 351)	282 (208, 336)	0.284
Albumin (g/dL)	4.0 (3.6, 4.3)	3.9 (3.3, 4.2)	4.1 (3.9, 4.3)	0.077
C-reactive protein (mg/dL)	0.32 (0.12, 1.21)	0.64 (0.17, 1.64)	0.15 (0.07, 0.55)	<0.001
Fasting blood glucose (mg/dL)	95 (90, 108)	94 (90, 101)	99 (93, 120)	0.046
Partial Mayo score	5 (4, 6)	6 (5, 7)	4 (3, 5)	<0.001
Mayo endoscopic subscore (n = 67)	2 (2, 2)	2 (2, 2)	2 (2, 2)	0.392
Previous treatment history				
5-ASA	97 (96.0)	56 (93.3)	41 (100.0)	0.144
PSL	28 (27.7)	0 (0.0)	28 (68.3)	<0.001
Immunomodulator	17 (16.8)	0 (0.0)	17 (41.5)	<0.001
AT	14 (13.9)	1 (1.7)	13 (31.7)	<0.001
Concomitant 5-ASA	85 (84.2)	50 (83.3)	35 (85.4)	1.000
Concomitant immunomodulator	7 (6.9)	1 (1.7)	6 (14.6)	0.017
Concomitant AT	15 (14.9)	1 (1.7)	14 (34.1)	<0.001

Values are presented as number (%) or median (interquartile range). PSL, prednisolone; BUD-MMX, budesonide multi-matrix; 5-ASA, 5-aminosalicylic acid; AT, advanced therapy.

**Table 2:** Treatment escalation and adverse events within 8 weeks in the overall cohort.

Outcome	Overall (n = 101)	PSL (n = 60)	BUD-MMX (n = 41)	*p* Value
Treatment escalation	27 (26.7)	16 (26.7)	11 (26.8)	1.000
Systemic corticosteroid intensification	12 (11.9)	9 (15.0)	3 (7.3)	0.355
AT initiation	15 (14.9)	7 (11.7)	8 (19.5)	0.393
Any adverse event	15 (14.9)	14 (23.3)	1 (2.4)	0.004

Values are presented as n (%). PSL, prednisolone; BUD-MMX, budesonide multi-matrix; AT, advanced therapy.

**Table 3:** Baseline characteristics of patients with baseline partial Mayo score 4–5.

Characteristic	PSL (n = 23)	BUD-MMX (n = 16)	*p* Value
Male sex	14 (60.9)	12 (75.0)	0.495
Age (years)	52 (37, 65)	46 (27, 60)	0.484
Duration of disease (months)	11 (3, 87)	55 (29, 80)	0.134
Disease extent			0.570
Proctitis	2 (8.7)	0 (0.0)	
Left-sided colitis	8 (34.8)	7 (43.8)	
Pancolitis	13 (56.5)	9 (56.3)	
Body mass index (kg/m^2^)	23.1 (19.7, 26.1)	21.9 (19.9, 25.3)	0.790
White blood cell count (/μL)	7500 (5100, 11,000)	6690 (5860, 7330)	0.575
Lymphocytes (/μL)	1542 (1298, 1905)	1381 (1224, 2042)	0.768
Hemoglobin (g/dL)	12.9 (11.0, 14.0)	13.1 (11.3, 14.4)	0.643
Platelet count (×10^3^/μL)	281 (223, 394)	287 (243, 360)	1.000
Albumin (g/dL)	4.0 (3.3, 4.2)	4.2 (3.6, 4.4)	0.159
C-reactive protein (mg/dL)	0.40 (0.12, 2.27)	0.08 (0.03, 0.31)	0.013
Partial Mayo score	5.0 (4.0, 5.0)	4.5 (4.0, 5.0)	1.000
Mayo endoscopic subscore (n = 23)	2 (2, 2)	2 (2, 2)	0.684
Previous treatment history			
5-ASA	21 (91.3)	16 (100.0)	0.503
PSL	0 (0.0)	10 (62.5)	<0.001
Immunomodulator	0 (0.0)	6 (37.5)	0.002
AT	1 (4.3)	4 (25.0)	0.139
Concomitant 5-ASA	20 (87.0)	12 (75.0)	0.415
Concomitant immunomodulator	0 (0.0)	2 (12.5)	0.162
Concomitant AT	1 (4.3)	5 (31.3)	0.033

Values are presented as n (%) or median (interquartile range). PSL, prednisolone; BUD-MMX, budesonide multi-matrix; 5-ASA, 5-aminosalicylic acid; AT, advanced therapy.

**Table 4:** Clinical and safety outcomes within 8 weeks in patients with baseline partial Mayo score 4–5.

Outcome	PSL (n = 23)	BUD-MMX (n = 16)	*p* Value
Clinical remission at week 4	11 (47.8)	7 (43.8)	1.000
Clinical remission at week 8	13 (56.5)	8 (50.0)	0.752
Clinical response at week 4	16 (69.6)	11 (68.8)	1.000
Clinical response at week 8	14 (60.9)	9 (56.2)	1.000
Treatment escalation	9 (39.1)	6 (37.5)	1.000
Systemic corticosteroid intensification	3 (13.0)	2 (12.5)	1.000
AT initiation	6 (26.1)	4 (25.0)	1.000
Any adverse events	5 (21.7)	0 (0.0)	0.066

Values are presented as n (%). PSL, prednisolone; BUD-MMX, budesonide multi-matrix; AT, advanced therapy.

**Table 5:** Unadjusted and CRP-adjusted logistic regression models for week 8 clinical remission in patients with baseline partial Mayo score 4–5.

	Unadjusted			CRP-Adjusted		
Characteristic	OR	95% CI	*p* Value	OR	95% CI	*p* Value
Treatment (BUD-MMX vs. PSL)	0.77	0.21–2.77	0.688	0.55	0.14–2.14	0.385
CRP	–	–	–	0.75	0.49–1.16	0.202

Values are odds ratios (ORs) from the logistic regression models. CRP, C-reactive protein; CI, confidence interval; OR, odds ratio.

## Data Availability

The deidentified data underlying this article are available from the corresponding author upon reasonable request and are subject to approval by the relevant institutional review board and applicable privacy regulations.

## Supplementary Materials

The following supporting information can be downloaded at: https://www.mdpi.com/article/10.3390/jcm15135115/s1, Table S1. Detailed adverse events observed within 8 weeks after treatment initiation; Table S2. Baseline characteristics and clinical outcomes in the sensitivity cohort.

## References

[1] Berre C.L., Honap S., Peyrin-Biroulet L. (2023). Ulcerative colitis. Lancet.

[2] Turner D., Ricciuto A., Lewis A., D’aMico F., Dhaliwal J., Griffiths A.M., Bettenworth D., Sandborn W.J., Sands B.E., Reinisch W. (2021). STRIDE-II: An Update on the Selecting Therapeutic Targets in Inflammatory Bowel Disease (STRIDE) Initiative of the International Organization for the Study of IBD (IOIBD): Determining Therapeutic Goals for Treat-to-Target strategies in IBD. Gastroenterology.

[3] Rubin D.T., Ananthakrishnan A.N., Siegel C.A., Barnes E.L., Long M.D. (2025). ACG Clinical Guideline Update: Ulcerative Colitis in Adults. Am. J. Gastro-Enterol..

[4] Raine T., Bonovas S., Burisch J., Kucharzik T., Adamina M., Annese V., Bachmann O., Bettenworth D., Chaparro M., Czuber-Dochan W. (2022). ECCO Guidelines on Therapeutics in ulcerative colitis: Medical Treatment. J. Crohns Colitis.

[5] Fardet L., Kassar A., Cabane J., Flahault A. (2007). Corticosteroid-induced adverse events in adults: Frequency, screening and prevention. Drug Saf..

[6] Feuerstein J.D., Rubin D.T., Aberra F.N., Yarur A.J., Malter L. (2025). Appropriate Use and Complications of Corticosteroids in Inflammatory Bowel Disease: A Comprehensive Review. Clin. Gastroenterol. Hepatol..

[7] Brunner M., Ziegler S., Di Stefano A.F., Dehghanyar P., Kletter K., Tschurlovits M., Villa R., Bozzella R., Celasco G., Moro L. (2006). Gastrointestinal transit, release and plasma pharmacokinetics of a new oral budesonide formulation. Br. J. Clin. Pharmacol..

[8] Maconi G., Camatta D., Cannatelli R., Ferretti F., Gabrielli A.C., Ardizzone S. (2021). Budesonide MMX in the Treatment of Ulcerative Colitis: Current Perspectives on Efficacy and Safety. Ther. Clin. Risk Manag..

[9] Sandborn W.J., Travis S., Moro L., Jones R., Gautille T., Bagin R., Huang M., Yeung P., Ballard E.D. (2012). Once-daily budesonide MMX^®^ extended-release tablets induce remission in patients with mild to moderate ulcerative colitis: Results from the CORE I study. Gastroenterology.

[10] Travis S.P., Danese S., Kupcinskas L., Alexeeva O., D’Haens G., Gibson P.R., Moro L., Jones R., Ballard E.D., Masure J. (2014). Once-daily budesonide MMX in active, mild-to-moderate ulcerative colitis: Results from the randomised CORE II study. Gut.

[11] Lichtenstein G.R., Travis S., Danese S., D’haens G., Moro L., Jones R., Huang M., Ballard E.D., Bagin R., Hardiman Y. (2015). Budesonide MMX for the induction of remission of mild to moderate ulcerative colitis: A Pooled Safety Analysis. J. Crohns Colitis.

[12] Rosiou K., San E.O.M., Kumar A., Esquivel K., Almas S., Stokes D., Ng T., Jayasooriya N., Ranasinghe I., Pollok R. (2021). Comparative outcomes of budesonide MMX versus prednisolone for ulcerative colitis: Results from a British retrospective multi-centre real-world study. J. Clin. Med..

[13] Osman A.M., Medhat M.A., Kamal G.M. (2025). Budesonide MMX versus prednisolone in mesalamine-refractory mild to moderate ul-cerative colitis: A randomized controlled clinical trial. Egypt. J. Intern. Med..

[14] Nakase H., Uchino M., Shinzaki S., Matsuura M., Matsuoka K., Kobayashi T., Saruta M., Hirai F., Hata K., Hiraoka S. (2021). Evidence-based clinical practice guidelines for inflammatory bowel disease 2020. J. Gastroenterol..

[15] Lewis J.D., Chuai S., Nessel L., Lichtenstein G.R., Aberra F.N., Ellenberg J.H. (2008). Use of the noninvasive components of the Mayo score to assess clinical response in ulcerative colitis. Inflamm. Bowel Dis..

[16] Akiyama S., Yokoyama K., Yagi S., Shinzaki S., Tsuruta K., Yoshioka S., Sako M., Shimizu H., Kobayashi M., Sakurai T. (2024). Efficacy and safety of filgotinib for ulcerative colitis: A real-world multicenter retro-spective study in Japan. Aliment. Pharmacol. Ther..

[17] Rubin D.T., Cohen R.D., Sandborn W.J., Lichtenstein G.R., Axler J., Riddell R.H., Zhu C., Barrett A.C., Bortey E., Forbes W.P. (2017). Budesonide multimatrix Is efficacious for mesalamine-refractory, mild to moderate ulcerative colitis: A randomised, placebo-controlled Trial. J. Crohns Colitis.

[18] Danese S., Hart A., Dignass A., Fiorino G., Louis E., Bonovas S., D’HAens G., Dotan I., Rogler G., Paridaens K. (2019). A multicentre prospective cohort study assessing the effectiveness of budesonide MMX^®^ (Cortiment^®^MMX^®^) for active, mild-to-moderate ulcerative colitis. United Eur. Gastroenterol. J..

[19] Maconi G., Mezzina N., Landi S., Grillo S., Bezzio C., Bosani M., Pastorelli L., Dell’ERa A., Chibbar R., Carmagnola S. (2019). Use, effectiveness and tolerability of budesonide-MMX in ulcerative colitis: A real-life experience. United Eur. Gastroenterol. J..

[20] Sandborn W.J., Danese S., D’Haens G., Moro L., Jones R., Bagin R., Huang M., Ballard E.D., Masure J., Travis S. (2015). Induction of clinical and colonoscopic remission of mild-to-moderate ulcerative colitis with budesonide MMX 9 mg: Pooled analysis of two phase 3 studies. Aliment. Pharmacol. Ther..

[21] Jesionowski M., Rydzewska G., Danese S., Paridaens K. (2023). Assessment of the effectiveness of Budesonide MMX^®^ for active, mild-to-moderate ulcerative colitis in the Polish sub-group of the CORE Practice prospective multi-centre observational study. Prz. Gastroenterol..

[22] Greenberg S., Herfarth H.H., Barnes E.L. (2019). Predictors of inadequate response to budesonide multimatrix in real-world patients with ulcerative colitis. Inflamm. Intest Dis..

[23] Bonovas S., Nikolopoulos G.K., Lytras T., Fiorino G., Peyrin-Biroulet L., Danese S. (2018). Comparative safety of systemic and low-bioavailability steroids in inflammatory bowel disease: Systematic review and network meta-analysis. Br. J. Clin. Pharmacol..

[24] Salice M., Rizzello F., Calabrese C., Calandrini L., Gionchetti P. (2019). A current overview of corticosteroid use in active ulcerative colitis. Expert Rev. Gas.-Troenterol Hepatol..

[25] Vasudevan A., Gibson P.R., Van Langenberg D.R. (2017). Time to clinical response and remission for therapeutics in inflammatory bowel diseases: What should the clinician expect, what should patients be told?. World J. Gastroenterol..

[26] D’Haens G. (2016). Systematic review: Second-generation vs. conventional corticosteroids for induction of remission in ulcerative coli-tis. Aliment Pharmacol. Ther..

[27] Walsh C., Ulaganathan H., Van Der Merwe K., Parihar V. (2025). P1139 A real-world, observational study comparing the side effects of budesonide MMX with prednisolone and the resolution of symptoms in patients with mild to moderate ulcerative colitis. J. Crohns Colitis.

