# Supplementary material for: Efficacy and Safety of Oral Prednisolone and Budesonide MMX for Outpatient Induction Therapy in Active Ulcerative Colitis: A Multicenter Retrospective Cohort Study
Source: J Clin Med. 2026 Jul 1;15(13):5115. doi: 10.3390/jcm15135115 (PMC13362799; doi:10.3390/jcm15135115)
Supplement: Supplementary file 1 [file jcm-15-05115-s001.zip › Supplementary_Table_S1_JCM revise.pdf]

**Supplementary Table S1. Detailed adverse events observed within 8 weeks after treatment initiation**

| Detailed adverse events | Overall (n=101) | PSL (n=60) | BUD-MMX (n=41) |
|---|---|---|---|
| **Neurologic events** | 3 | 3 | 0 |
| Seizure | 2 | 2 | 0 |
| Spinal cord infarction* | 1 | 1 | 0 |
| **Psychiatric events** | 2 | 2 | 0 |
| Psychiatric symptoms/disorder | 1 | 1 | 0 |
| Depression | 1 | 1 | 0 |
| **Dermatologic events** | 4 | 4 | 0 |
| Rash | 2 | 2 | 0 |
| Pruritus | 1 | 1 | 0 |
| Eczema | 1 | 1 | 0 |
| **Infectious events** | 2 | 1 | 1 |
| COVID-19 | 1 | 1 | 0 |
| Bronchopneumonia | 1 | 0 | 1 |
| **Other events** | 4 | 4 | 0 |
| Chest pain | 1 | 1 | 0 |
| Facial edema | 1 | 1 | 0 |
| Lower limb edema | 1 | 1 | 0 |
| Stomatitis | 1 | 1 | 0 |

Data are shown as the number of events.

* Serious adverse event. PSL, prednisolone; BUD-MMX, budesonide MMX.
