# Supplementary material for: Efficacy and Safety of Oral Prednisolone and Budesonide MMX for Outpatient Induction Therapy in Active Ulcerative Colitis: A Multicenter Retrospective Cohort Study
Source: J Clin Med. 2026 Jul 1;15(13):5115. doi: 10.3390/jcm15135115 (PMC13362799; doi:10.3390/jcm15135115)
Supplement: Supplementary file 1 [file jcm-15-05115-s001.zip › Supplementary_Table_S2_JCM revise.pdf]

Supplementary Table S2. Baseline characteristics and clinical outcomes in the sensitivity cohort

Characteristic	PSL subgroup (n=31)	BUD-MMX (n=41)	P value
Male sex	17 (54.8)	24 (58.5)	0.813
Age (years)	50 (32.5, 60.5)	54 (30, 66)	0.923
Duration of disease (months)	16 (2.5, 52)	77 (27, 134)	<0.001
**Disease extent**			0.391
Proctitis	2 (6.5)	1 (2.4)	
Left-sided colitis	6 (19.4)	13 (31.7)	
Pancolitis	23 (74.2)	27 (65.9)	
Body mass index (kg/m²)	21.6 (19.7, 24.5)	21.6 (19.6, 23.9)	0.597
White blood cell count (/μL)	7500 (6085, 10090)	6040 (5190, 7275)	0.017
Lymphocytes (/μL)	1630 (1278, 1910)	1582 (1225, 2070)	0.931
Hemoglobin (g/dL)	12.5 (11.3, 13.6)	13.0 (11.3, 13.9)	0.688
Platelet count (×10³/μL)	305 (242, 356)	282 (208, 336)	0.391
Albumin (g/dL)	3.6 (3.3, 4.1)	4.1 (3.9, 4.3)	0.004
C-reactive protein (mg/dL)	0.65 (0.23, 1.63)	0.15 (0.07, 0.55)	0.002
Fasting blood glucose (mg/dL)	95 (90, 101)	99 (93, 120)	0.113
Partial Mayo score	6 (5, 7)	4 (3, 5)	<0.001
Mayo endoscopic subscore (n=45)	2 (2, 2)	2 (2, 2)	0.632
**Previous treatment history**			
5-ASA	30 (96.8)	41 (100.0)	0.431
PSL	0 (0.0)	28 (68.3)	<0.001
Immunomodulator	0 (0.0)	17 (41.5)	<0.001
AT	1 (3.2)	13 (31.7)	0.002
Concomitant 5-ASA	25 (80.6)	35 (85.4)	0.751
Concomitant immunomodulator	0 (0.0)	6 (14.6)	0.033
Concomitant AT	1 (3.2)	14 (34.1)	0.001
**Clinical outcomes, treatment escalation, and adverse events**			
Clinical remission at week 4	15 (48.4)	18 (43.9)	0.812
Clinical response at week 4	25 (80.6)	24 (58.5)	0.073
Clinical remission at week 8	20 (64.5)	22/40 (55.0)	0.472
Clinical response at week 8	23 (74.2)	25/40 (62.5)	0.320
Treatment escalation within 8 weeks	8 (25.8)	11 (26.8)	1.000
Systemic corticosteroid intensification	2 (6.5)	3 (7.3)	1.000
AT initiation	6 (19.4)	8 (19.5)	1.000
Any adverse event within 8 weeks	7 (22.6)	1 (2.4)	0.018

Values are presented as number (%) or median (interquartile range). PSL, prednisolone; BUD-MMX, budesonide multi-matrix; UC, ulcerative colitis; 5-ASA, 5-aminosalicylic acid; AT, advanced therapy
